# Applying to medical school with undiagnosed dyslexia: a collaborative autoethnography

**DOI:** 10.1007/s10459-023-10258-3

**Published:** 2023-07-10

**Authors:** Megan Cornwell, Sebastian Charles Keith Shaw

**Affiliations:** 1 Department of Medical Education, Brighton and Sussex Medical School, 344B Watson Building, Falmer, BN1 9PH England; 2 Lecturer in Medical Education (Research Methods), Department of Medical Education, Brighton and Sussex Medical School, 344B Watson Building, Falmer, BN1 9PH England

**Keywords:** Dyslexia, Autoethnography, Undergraduate medical education, Admissions process, Medical students

## Abstract

Recent statistics found the prevalence of dyslexia in UK medical schools to be 7%, sitting below the national prevalence of 10%. The factors contributing to this discrepancy are currently unknown, but may result from an interplay of individual and systemic barriers to entering medicine. This collaborative, analytic autoethnography aimed to use the experiences of ‘Meg’, a fourth-year medical student who was diagnosed as dyslexic whilst at medical school, to explore how the lack of a diagnosis during the admissions process may have impacted her journey into medicine. The data were collected using reflective writing and an interview before conducting a thematic analysis. Our analysis resulted in the construction of two meta-themes, relating to the negative emotional impact of not having a diagnosis and feelings of inferiority. Seven themes were also constructed. Some explored how Meg’s personal experience of undiagnosed dyslexia acted as a barrier to entering medicine. Others explored the impact of external factors, such as socio-economic background and the provision of support, on an individual’s chance of successfully applying to medical school. Finally, we explored the inadvertent impact undiagnosed (and unrecognised) dyslexia had on Meg’s life course, including how medicine-specific aptitude tests, such as the BMAT and UKCAT, may have contributed to this. These results provide a unique window into the culture of applying to medical school as an undiagnosed dyslexic person, whilst discussing the need for medical schools to consider how their admissions processes may inadvertently disadvantage undiagnosed dyslexic applicants.

## Introduction

Through a medical lens, dyslexia may be defined as a ‘specific learning difficulty’ (SpLD) associated with differences in reading, writing, and spelling, despite a ‘normal intelligence’, intact sensory abilities, and adequate education (Peterson & Pennington, 2012; Peteretto & Masala, 2017). However, there is currently no globally accepted definition, creating issues with diagnostic consistency (Peteretto & Masala, 2017). These are further compounded by dyslexia existing as a continuum, meaning that the impact of differences on day-to-day life within current social and environmental expectations varies between individuals and contexts (Peteretto and Masala, 2017; Miles et al., 2003). Dyslexia may also manifest as slow writing, poor organisational skills, poor time management and poor short-term memory (Peterson and Pennington, 2012). All of these have the potential to impact an individual’s ability to cope in higher education settings – especially one as demanding as medicine. However, under the neurodiversity paradigm, it is vital to consider that strengths and challenges are highly contextual, depending on external factors and subsequent impacts on individual differences (Shaw et al., 2021).

Medical students and doctors have described various challenges linked to being dyslexic, either due to personally experienced difficulties or due to peers’ attitudes (Shaw et al., 2016; Anderson & Shaw, 2020; Newlands et al., 2015; Shaw et al., 2019; Hennessy et al., 2020). Reported struggles include anxiety and stress around performance, difficulties with prescribing, handover, and ward rounds (Newlands et al., 2015; Shaw et al., 2019). However, in line with the aforementioned neurodiversity paradigm, the dyslexic neurotype can also bring many strengths. For example, previous studies have reported that dyslexic medical students and doctors self-identify as being more empathetic, having stronger communication skills, and being better teachers than their non-dyslexic counterparts, whilst broader studies on neurodivergent medical students have identified strengths such as holistic attitudes to care, attention to detail, and creative thinking (Shaw et al., 2016; Shaw & Anderson, 2018; Rowlands et al., 2013; Shaw et al., 2023). This reinforces the importance of adjusting external factors – e.g., social, cultural, or environmental – in an effort to ameliorate disablement and, where possible, to empower differences to instead manifest as strengths.

Dyslexia was previously referred to as a ‘learning disability’. Two theoretical models can be used to describe how disability is viewed in society: the medical and the social models (Haegele & Hodge, 2016). The main difference between the two is how disability is viewed as either a problem at an individual or a societal level (Haegele & Hodge, 2016; Terzi, 2004). The medical model views disability as when an individual has a physical or mental deficit and is deeply connected with the rise of the medical profession within society (Haegele & Hodge, 2016). This model seeks to treat this perceived ‘deficit’, to allow disabled people to gain independence and function within society, placing the onus upon the individual to conform and adapt. In the past, those who were unable to be ‘treated’ were often segregated from society through the use of institutions (Haegele & Hodge, 2016). This model is critiqued for its inability to recognise the impact of the sociocultural environment within which people are situated on the level of disability experienced (Haegele & Hodge, 2016). However, the medical model is still very present within society, with medical professionals often acting as the gatekeepers to a diagnosis, alongside the treatment or support this brings (Haegele & Hodge, 2016). Where the medical model depicts disability as a problem at an individual level, the social model depicts the problem as being at a societal level (Haegele & Hodge, 2016). In the social model, a disability is instead referred to as an ‘impairment’. An individual with an ‘impairment’ becomes disabled when barriers within society act to exclude them because of their impairment (Haegele & Hodge, 2016; Terzi, 2004). This approach places the onus upon society to make modifications that allow those with ‘impairments’ to gain independence and participate as active members within it (Terzi, 2004; Oliver, 2013). Legislation such as the United Kingdom (UK) Equality Act (2010) reflects a societal shift from viewing disability through the lens of the medical model to the social model, using the law to enact societal changes that help to remove disabling barriers (Terzi, 2004; Oliver, 2013).

Given its long-lasting nature and potential to impact day-to-day life, dyslexia may be considered a disability, which is a protected characteristic under the UK Equality Act (2010). This label stops education providers, including higher education centres, and therefore medical schools, from discriminating between candidates based on dyslexia – whilst also requiring them to make reasonable adjustments to allow dyslexic students to achieve their full potential (Equality Act, 2010). The current literature suggests that dyslexic medical students underperform compared to their peers in the early years of medical school, but this difference then disappears later in their course if they receive adequate supports (Shaw et al., 2017). This is in keeping with the social model of disability and demonstrates that dyslexic people should be given the same chance to enter medical school as other candidates through reasonable adjustments. In doing so, we can also recognise the benefit that dyslexic doctors may bring for dyslexic people accessing healthcare.

## Aims

A discrepancy exists between the prevalence of dyslexia in medical students compared with the general population, at seven and ten% respectively (Angell et al., unpublished). Whilst previous literature has identified factors such as low self-esteem and lack of support as barriers for dyslexic students to higher education, there are potentially additional factors contributing to this discrepancy, including issues with disclosure, under-diagnosis, and/or a discriminatory medical school admissions process (O’Byrne et al., 2019). Medicine is a unique and demanding course and is therefore likely to have specific barriers which have yet to be identified and explored at either an individual or systemic level. This study aims to utilise the experience of a fourth-year medical student of applying to medical school with undiagnosed dyslexia to explore the potential barriers that could prevent or deter those with undiagnosed (or unrecognised) dyslexia from entering medical schools in the UK, and how those barriers may relate to the larger societal issues surrounding widening access to medicine.

## Methods

### Researcher positionality

#### MC (“Meg”)

*I sat in my lecture on dyslexia in medical education, astounded by how much I was learning about myself and the difficulties I experience, shocked at how little I knew despite being diagnosed nearly four years prior. I sat in bemused wonder at the impact dyslexia had had upon my life without my knowledge, having never quite understood the extent to which it can affect an individual before this point. I kept having moments of understanding as to why I found particular tasks difficult and found myself reflecting upon how the United Kingdom Clinical Aptitude Test (UKCAT) seemed to target many of the difficulties that resonated with me. I wondered how else my dyslexia may have impacted my life in ways I had never before realised. (Vignette 1).*

**Fig. 1:**
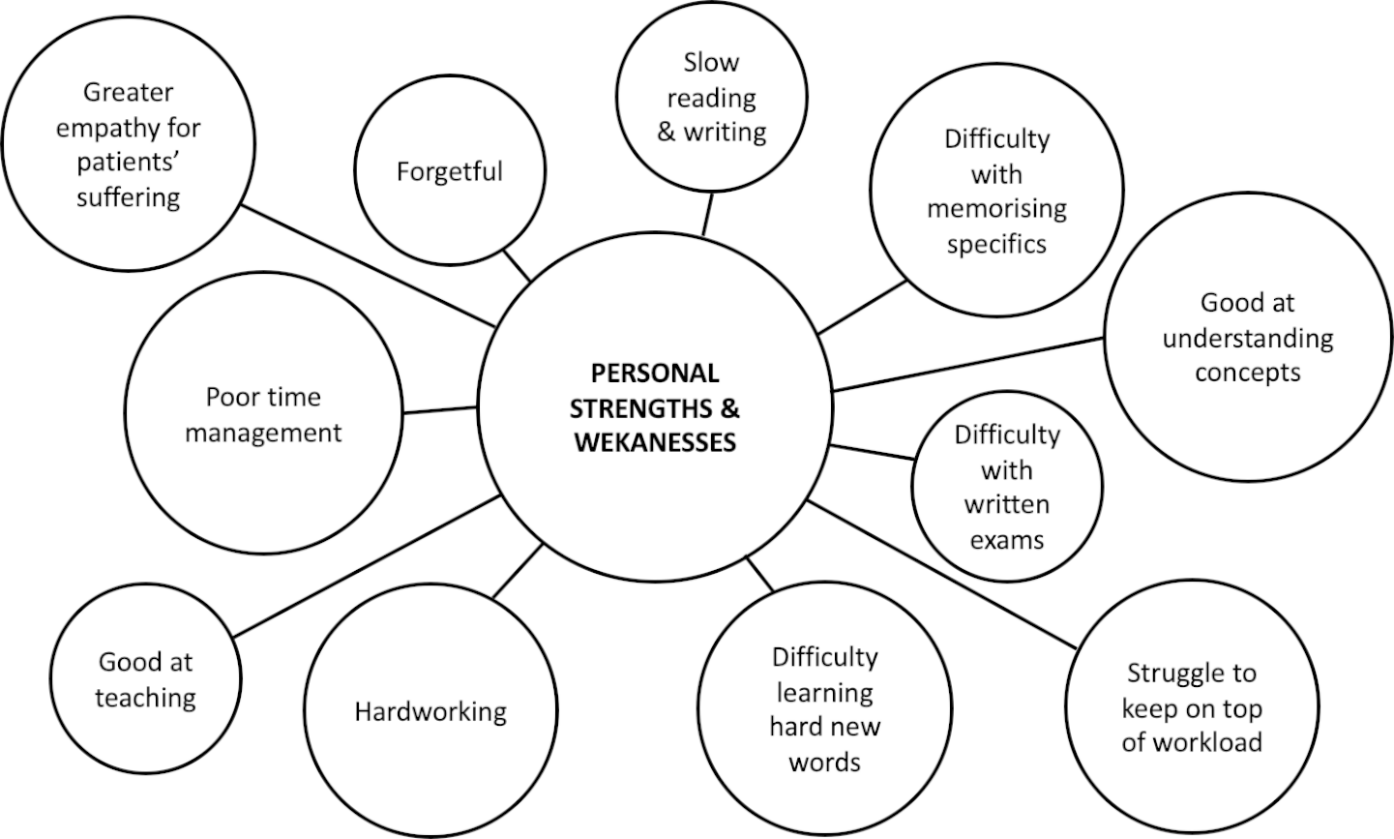
Illustration of Meg’s personal strengths and weakness that she associates with her dyslexic profile

I am a fourth-year medical student at Brighton and Sussex Medical School. Like the majority of dyslexic doctors in the UK, I was diagnosed as dyslexic after starting medical school and am just now beginning to understand my dyslexia (Fig. 1) (Anderson & Shaw, 2020). Therefore, my experience of the medical school admissions process was one with undiagnosed dyslexia–as someone struggling with dyslexic difficulties without the support that a diagnosis brings. My secondary and further education consisted of two quite different schools, a comprehensive school for my secondary education and a grammar school for my further education, which gave me an insight into how different schools approach dyslexia. Early in my further education I decided to pursue a career in medicine, which heavily influenced my life decisions from that point– everything revolved around applying to medical school.

The highly competitive nature of medical school applications meant that I decided to take a gap year soon after deciding to pursue a career in medicine to strengthen my application. However, I did not achieve the required grades, meaning that I had to retake, which restricted the medical schools I could apply to in the UK. Fortunately, I was accepted into one of the three medical schools that I could still apply to; however, my mid-lecture reflections have left me contemplating how an earlier diagnosis may have affected this and whether others have had similar experiences to my own.

#### SS (“Seb”)

I am a Lecturer in Medical Education (Research Methods) at Brighton and Sussex Medical School. I am also a doctor working in the UK National Health Service. Much like Meg, I am also dyslexic. In fact, I am ‘multiply neurodivergent’, in that I am dyslexic, but I am also autistic and ADHD. Through my lived experience, I advocate for the neurodiversity paradigm and believe these neurotypes to be differences, not disorders, nor inherent impairments. I teach the dyslexia session Meg discusses in vignette 1, which is how we first met.

I identify as a social constructionist and have previously researched various facets of the experiences of neurodivergent medical students and doctors – including those identifying as dyslexic (Shaw et al., 2016; Shaw et al., 2018; Shaw et al., 2022), dyspraxic (Walker et al., 2018; Walker et al., 2021), and autistic (Shaw et al., 2023). I am passionate about empowering fellow neurodivergent people to undertake research in this area, helping to shape the evolving discourse directly. Through doing this, we can hope to tackle the longstanding epistemic injustice that accompanied the medical model, reclaiming our status as credible creators of knowledge about ourselves, our neurotypes, and our experiences of the world around us.

I have a particular interest in qualitative methods and teach these on the postgraduate courses at the medical school. Of particular relevance to this study, I have used collaborative autoethnographies to explore the experience of studying medicine as a dyslexic or dyspraxic person (Shaw et al., 2016; Walker et al., 2020), and on supporting dyslexic doctors with prescribing (Shaw et al., 2022). Having previously adopted both primary researcher and supportive roles in autoethnographies, I was able to support and guide Meg through her own autoethnographic journey. We met regularly on Microsoft Teams throughout the development of this research paper. This allowed us to catch up personally and academically each week, which is an essential part of the autoethnographic process, given its associated “visibility” and potential to make one feel vulnerable.

Finally, it is also worth considering another aspect of my positionality here. You will see in our results how Meg reflects on the positive impact of the Army Cadet Force during her struggles with further education. I am currently a Captain in the Army Cadet Force and have spent many years reflecting on the positive impacts it had on me – both as a cadet and, later, as an instructor and officer. Whilst we did not know one another back then, Meg and I were involved in cadets at the same time, albeit myself as an officer and her as a cadet. Given this unexpected link, I felt well placed to help support Meg’s reflection in this specific area too when it arose.

### Approach and philosophy

Due to the challenging nature of exploring the intricate and often intangible sociocultural factors that produce the aforementioned barriers, we chose to use a qualitative approach for this project. This study is an autoethnography that takes relativist (ontological) and subjectivist (epistemological) stances. Within subjectivism, reality is considered to be constructed by the mind; thus, knowledge is constructed internally, with the meaning being imposed on an object (Al-Ababneh, 2020; Holden, 2004). The interpretivist research paradigm, rooted in a subjectivist epistemology, is based on the idea that our interpretation of reality is influenced by sociocultural factors and historical context (Al-Ababneh, 2020). Interpretivism treats each situation as unique and aims to understand the complexity of the relational and contextual factors (Holden, 2004). Researchers within this paradigm typically use qualitative approaches, which seek to explore specific and complex phenomena through immersion, interpretation and analysis of an environment (Holden, 2004).

### Autoethnography

Autoethnography synergistically combines autobiography with ethnography (Adams et al., 2017). To understand autoethnography, it is essential to first understand ethnography. Ethnography is a qualitative methodology in which the researcher immerses themselves within a culture to better understand the cultural, societal and interpersonal factors influencing the experiences of the individuals within it (Adams et al., 2017). Autoethnography is similar in its aims and therefore has roots in a subjectivist epistemology (Farrell et al., 2015; Holden, 2004). Autoethnography assumes that we are each complete participants in our own social and cultural realities. As such, our life stories represent full immersion in this culture. Thus, researchers can use their own experiences to shed light on particular aspects of their culture (Adams et al., 2017; Farrell et al., 2015). The power of autoethnography stems from its ability to push for social justice through shedding light on institutional cultures (Ngunjiri, 2010; Grant, 2019). It not only seeks to provide rich information on the experiences but also to alter or challenge harmful perceptions held by cultural outsiders (Adams et al., 2017; Ngunjiri, 2010; Grant, 2019). As a methodology, autoethnography embraces subjectivity, critically reflecting upon the impact of researchers’ emotional responses, not only within the life stories explored, but also within the autoethnographic process itself (Farrell et al., 2015). To that end, this methodology is considered to have therapeutic benefits for both researchers and readers (Ellis et al., 2011).

Two main schools of autoethnography exist: analytic and evocative (Anderson, 2006). Evocative autoethnography uses rich description to evoke an emotional response and make the researcher’s account of their experience resonate with readers (Ellis & Bochner, 2006). This approach focuses on storytelling, embracing the individuality of each experience (Ellis & Bochner, 2006). Analytic autoethnography steps beyond this to use the researcher’s experiences to explore and create or apply sociocultural theories. This methodology places a greater emphasis on analysis and how the results may be transferable beyond the researcher’s own story (Anderson, 2006; Ellis & Bochner, 2006). The systematic approach to data collection and analysis in analytic autoethnography makes it best suited to the current body of medical education literature (Farrell et al., 2015).

The specific methodology used in this study is that of a collaborative, analytic autoethnography (Chang et al., 2012). Whilst this study focused upon Meg’s experiences, by having a second researcher, and therefore an outside perspective to Meg’s specific story, actively involved in the data collection and analysis, we aimed to achieve greater depths of personal interrogation (Chang et al., 2012). For this study, the collaborative approach was also necessary as Seb’s expertise with autoethnographic methodology ensured the use of correct methodology despite Meg’s inexperience.

### Ethical considerations

This study was approved by the Brighton and Sussex Medical School Research Governance and Ethics Committee. As this research project required deep introspection on both being dyslexic and the experiences and challenges that resulted from this, there was a real potential for Meg to experience psychological distress and a sense of vulnerability. However, Seb was well versed in both the subject matter and methodology and was therefore aware of this possibility. Subsequently, he fostered psychological safety through a level hierarchy and regular check-ins in with Meg. Should they be required, appropriate signposting for support was also mutually agreed in advance of the study. In addition to considering Meg’s own well-being, it was also important to consider the implications for those who played a role in her story – in the words of Lapadat (2017), the ‘implicated others’. Those she had interacted with during the period of her life discussed in this project are interwoven into her story, acting to create and alter her experiences, and thus would be difficult to remove. However, it is challenging to gain consent from everyone mentioned. As such, it is standard practice within autoethnographic research to alter certain fine details, whilst maintaining the central messages and emotional rawness (Shaw et al., 2022). Our study has embodied such relational ethical principles in this way, striving to protect the identities of these implicated others (Shaw, 2019).

### Data collection

Our approach was twofold. Initially, Meg wrote a reflective account recounting her own experiences of the medical school admissions process with undiagnosed dyslexia. As is common with an analytic autoethnographic approach, during this initial writing process Meg engaged in discussions with others, both from and after this time period, during the writing process to avoid self-absorption and use the additional perspectives to enhance the reflective process (Anderson, 2006). From this reflective piece, an interview topic guide was produced in an iterative process between Meg and Seb. This aimed to explore issues in greater depth and cover areas of potential interest that were not covered in the initial reflection. Seb then used this to conduct a loosely structured interview with Meg over Microsoft Teams. This was audio-recorded.

### Data analysis

First, Meg transcribed the interview recording verbatim, which helped with further re-immersion in her own story. We then thematically analysed each data source independently in the first instance. This followed the six-phase framework for thematic analysis of Braun and Clarke (2006). Initially, we immersed ourselves in the data. This was aided by the transcription process. We then began with the manual, open coding of the data. Preliminary themes were then constructed to encompass codes relating to similar concepts. We reviewed our initial themes together and decided upon the final themes in an iterative manner, relating each one back to the original data to ensure they remained a true representation. During this step, the themes for each data source were combined due to their undeniable similarities. Once the final themes were decided upon, we discussed and defined the specifics of each theme, ensuring they were ready for the write-up.

### Narrative structure

Whether analytic or evocative, the power of autoethnography lies in the narrative (Anderson, 2006; Ellis & Bochner 2006). Whilst research often conforms to the standard structure of Introduction, Methods, Results and Discussion, the rigidity of this format disrupts the narrative and is therefore ill suited to the reporting of autoethnographies (Shaw et al., 2018). As such, the results shall be presented interwoven with the discussion, allowing for a greater sense of the narrative to be created and remaining true to the methodology. We also incorporate vignettes throughout. These provide rich immersion into Meg’s culturally grounded experiences and are common practice within reports of autoethnographic research. As such, much of our results are also presented in the first person, giving voice to Meg’s experiences.

## Results

I don’t know whether a diagnosis would have given me access to additional support back then, but it may have. It may have emboldened me to ask for support as it would have validated why I needed it when others didn’t. If I had, maybe I would have passed maths the first time around and gotten the three A’s needed to apply to most medical schools in the UK.

Our analysis resulted in the construction of seven themes and two persistent meta themes (Table 1). These related to the negative emotional impact of being undiagnosed (and mostly unrecognised) as dyslexic during the medical school admissions process, and feelings of inferiority. Each of these permeated all other themes.

**Table 1:** Overview of findings

THEMES			KEY FINDINGS
Negative Emotional Impact	Feelings of Inferiority	The self as a barrier	- My personal perception of dyslexia which was built on societal stigma and own experience - The negative emotional response to a potential diagnosis - My perceived inability to ask for help
		Impact of being undiagnosed as dyslexic during education	- My individual difficulties with slower reading and writing speeds and issues with misreading - The importance of developing appropriate exam technique - The necessity of having a non-academic outlet for developing my confidence
		Support	- The importance of support being sufficient and early - The onus being placed upon the home network to be the main providers of support - The importance of the provision of appropriate support at all levels of academic ability - “Masking” as a barrier to support - The benefits of support at an institutional level
		Socio-economic barriers	- 3 A-grades are not always equal - Candidate’s backgrounds may be compounding the difficulties experienced because of undiagnosed dyslexia - The impact of an individual’s socio-economic background upon the support available - The different priorities of different types of school
		Personal impact of medical school admissions process	- The requirement for an early commitment to academic success - The emotional impact of the pressure placed upon a candidate
		What makes a good doctor	- The importance of academic ability vs. the desired personality characteristics - How good is the current selection process in selecting the best candidates?
		Undiagnosed dyslexia influencing life course	- The limitations of which medical schools were available to me based on my retakes and UKCAT score - My lack of control over my choices - The retrospective mitigating circumstances that a diagnosis would have brought

### The self as a barrier

During my work experience, I did a week of working in a shop. Telling customers how much they had to pay for items highlighted how often I was misreading numbers, where I was either having to correct myself or the customer would question the price and I would realise my mistake. One such customer pointed out my mistake and, on apologising, he replied “don’t worry, I have number dyslexia too”. This comment took me completely by shock, and I felt like it struck a chord within me. However, this frightened me, and I pushed this feeling aside, going into denial about the fact that this statement may have been true and refusing to question why someone thinking I was dyslexic affected me..

These feelings of denial and fear at a potential diagnosis were something that I experienced at different points throughout my education. Previous research has shown that I am not alone in this experience, with a negative stereotype of dyslexia and the perception of peers often identified as factors driving this response (Shaw et al., 2016; Livingston et al., 2018). This negative emotional response acted as an internal barrier to exploring the potential of receiving a diagnosis and, therefore, accessing additional support. On reflection, these feelings were driven by my perception of what being dyslexic meant, which was built upon my experiences of dyslexic people around me, as well as the wider societal stigma (Livingston et al., 2018; Macdonald, 2010).

This image of dyslexia meaning lower intelligence and finding reading difficult both scared me from trying to seek help and helped me to convince myself that I could not be dyslexic.

My perception of dyslexia remained largely negative despite having an increased awareness of dyslexia through friends and family. Those with less awareness and familial acceptance may associate dyslexia with a greater stigma and therefore be more fearful of a diagnosis, making both the home and school environments potential barriers to seeking a diagnosis (Livingston et al., 2018; Macdonald, 2010).

The teachers were so used to teaching to those people as well, that understand so quickly, that they don’t really slow down for anyone else, and I just felt like I could not ask for help…

In a school where I considered everyone around me to be considerably more intelligent and where they were able to grasp these concepts with relative ease, I could barely acknowledge how much I was struggling…

My self-perception and emotions also acted as barriers to asking for additional support. Whilst I was unaware that my academic struggles were due to unrecognised and unsupported dyslexia, I was aware that I struggled more than my peers during my A-level (further education) years. The feelings of inferiority this created built an internal barrier to simply asking my peers or teachers for help. I was embarrassed that I could not do what others appeared to do with ease. Undiagnosed dyslexics are often stereotyped as lazy or stupid prior to their diagnosis (Livingston et al., 2018). During this time, I believed this of myself, thinking I was lazy and just needed to work harder – I did not deserve help.

Through the lens of the social model of disability, these personal barriers stopped me from seeking additional support and, subsequently, created an increasingly disabling environment. This ultimately contributed to not achieving the required grades and restricted which medical schools were available to me based on my academic performance.

*My heart sank at the large red ‘D’ at the top of my physics test. I looked across at my friend’s ‘B’ and felt even worse. It had been a hard test, but I thought I had done enough to at least pass. “Oh well”, I said, trying to laugh it off. “I didn’t really revise, and who cares about physics anyway!” But I’m lying, I do care. With only months until the final exams, I wonder how on earth I’m ever going to understand enough physics to get the grades I need. Maybe I won’t and maybe I need to accept that I’m just not good enough to get into medicine. My mind wanders to mental health nursing again. I like mental health, I’d still be caring, and I might actually stand a chance of getting in. Maybe I’ll have another look at the entry requirements for mental health nursing later… (Vignette 2).*

A diagnosis in adulthood, which many dyslexic doctors experience, is associated with low self-esteem and low self-confidence (Anderson & Shaw, 2020; Livingston et al., 2018). This poor self-perception may limit individuals’ aspirations (Livingston et al., 2018). Many potential doctors may never consider medicine a viable career option because they do not receive an early diagnosis or appropriate support. Whilst my moments of low self-confidence ultimately did not deter me from pursuing a career in medicine, there were times when I questioned my ability and considered pursuing less competitive careers with lower grade requirements, such as teaching and mental health nursing. Without the strong support network I had to encourage me, I would likely have given up on the fantasy of medicine.

### Impact of being undiagnosed as dyslexic during education

I noticed at a young age that I was slower than my peers, requiring more time to read, to write, to understand new concepts and, most importantly, to finish exams. I also noticed that I would regularly misread words and numbers. This was highlighted when I started doing exams – I often got answers wrong due to misreading questions. This misreading was worse when under stress or time-pressure, which added an additional challenge to exams.

*I flicked through the remainder of the exam paper in despair. Too many questions left to even count and too few minutes left to answer them*. *How did I always seem to end up in this situation? My hand was painful from writing so fast, trying desperately to get any answers possible down on the paper. Skim the question, skim it again to make sure I’ve actually read it. I’m still not sure what I’ve read, so I read it again, slower. I don’t have time for this! Skip that question and onto the next. I can feel the adrenaline and the panic setting in, my foot is shaking wildly in response. “Just keep writing”, I tell myself, “just keep writing”. I don’t finish the paper. Again.* (Vignette 3).

Over the years, with additional help from my family, I developed exam techniques to partially compensate for these differences. These included straightforward methods, such as finger-pointing whilst reading, re-reading questions, and writing out numbers to ensure I had read them correctly. With the help of these compensatory techniques, I was able to improve my performance in written exams. However, these techniques all required additional time, adding to the time pressure I already felt. Whilst these techniques proved to be vital in my ability to survive written exams, they were not transferable to other exam formats, such as those conducted on computers.

The challenges that I faced academically, which became more challenging as I progressed through the education system, led me to want an area of my life that was not based on academia. I needed a distraction from that painful aspect of my life.

I think army cadets was actually such a good outlet for me at the time, cause even when I was really frustrated, I had that to like escape to… It was so nice and simple, and it was stuff that I could understand so easily. I loved army cadets.

For me, the Army Cadet Force offered a non-academic outlet to which I could apply myself. Succeeding in this area and being pushed to become both a teacher and a leader helped improve my self-confidence. This sense of self-belief likely acted to combat the low self-confidence I felt in my school life and helped to give me the confidence to pursue a career in medicine.

### Support

The extent of the difficulties that dyslexic individuals experience varies significantly, meaning the support required is person-specific (Alexander-Passe, 2017; Miles et al., 2003). Under the social model of disability, the provision of additional support should, in theory, allow dyslexic people to perform on par with their peers (Oliver, 2013). However, without this support, dyslexic individuals are disadvantaged, creating additional barriers to medical school beyond those created by the highly competitive nature of the admissions process.

I think it must be so easy to lose faith in yourself… So easy to think ‘oh, I just can’t do this’, and then you stop trying, because actually, it’s really hard to keep trying and trying and failing every time. That’s got to be so demoralising… If you don’t have that support, from anywhere, family, teachers, friends… you don’t get the right GCSEs (secondary education grades) to go and do the A-levels (further education) you need, you suddenly can’t even consider that you could go to medical school… It’s really sad, because I bet those people would be amazing doctors if they really wanted to be. Or would be amazing at whatever they wanted to do, and it’s just that they didn’t have the right support.

My experiences reiterate the need for support to be appropriate and provided from an early point to ensure that early failure does not prevent future opportunities. This is especially important as the literature suggests that those who receive a late diagnosis are likely to doubt their abilities and lose motivation (Livingston et al., 2018). My experience was one of the onus being placed upon an individual’s home support network to be the primary providers of this additional support, be that through encouragement or the provision of additional tuition. My personal experience was one of privilege, where my parents were able to support me in a variety of ways. They encouraged me to not give up after the failures that I experienced and paid for additional tuition after struggling with chemistry. This placement of responsibility upon individuals’ home support networks creates immediate socioeconomic disparities.

Another issue is the requirement of a diagnosis to access support. The terms “masking” or “camouflaging” are widely accepted within the neurodiversity literature, more specifically that relating to autism (Hull et al., 2017; Pearson & Rose, 2012). This describes an individual using specific behaviours and coping mechanisms to obscure their differences from society. This concept can also be applied to dyslexia (Alexander-Passe, 2017).

I think that one of the biggest things that helped to mask my dyslexia… is the assumption that if reading is more difficult for someone, then they would enjoy it less and not actively seek it out. I was the opposite of this, an absolute bookworm, and always have been from a young age. At school, I was a regular at the library and was constantly moving from one book to the next.

Recognition of masking within the undiagnosed dyslexic community is important (Alexander-Passe, 2017). Within the autistic community, masking is associated with adverse long-term outcomes, such as exhaustion, anxiety and low self-esteem (Hull et al., 2017). Dyslexic adults likely experience similar long-term outcomes and yet, without a diagnosis, are likely to have no explanation for these. The ability to unintentionally hide dyslexia is likely due to the current lack of awareness at a societal level, combined with stereotyping of dyslexic people as being unintelligent (Livingston et al., 2018). Masking, combined with the difficulties of receiving a diagnosis due to the lack of a definitive definition, illustrates how a dyslexic person might never receive a diagnosis and, therefore, never receive the additional support that a diagnosis would bring. Those with the power to diagnose acting as gatekeepers to support is one of the issues of the medical model of disability remaining prominent within society (Haegele & Hodge, 2016).

One method my schools used to provide additional support was to provide study skills workshops for everyone. This approach to support at an institutional level ensured that those who were still performing well, but may be disadvantaged by a hidden SpLD, still received support. Whilst support on an individual level would likely be more beneficial, support at an institutional level is a step in the right direction in large schools where this is difficult to provide.

### Socio-economic barriers

Having highlighted the benefits of early support, it is important to discuss the societal factors that may impact the level of support received. To explain the importance of societal factors and understand their relationship to undiagnosed dyslexia, one must consider the challenges I faced, despite my position of privilege, and how these might be compounded by socio-economic barriers to support.

I was fortunate, my parents could afford a tutor for me in chemistry, so, that really, really helped… It helped me catch up and helped me understand the second-year stuff.

As discussed earlier, my struggles with chemistry at a higher level led my parents to hire a chemistry tutor to ensure that failure in chemistry would not prevent me from pursuing a career in medicine. A tutor was only an option due to the privilege of my parents being able to afford this, which allowed me to achieve the required grade. Without this, I could not have applied to study medicine as an undergraduate. Whilst postgraduate applications are possible, less financial support is available for postgraduate study, which may act to further prohibit medical training for those from lower socio-economic backgrounds.

Factors such as socio-economic background have the potential to influence the impacts of undiagnosed dyslexia. This could be through lack of adequate support or by reducing the amount of time available to spend on schoolwork, despite requiring more time to complete it. Even receiving a diagnosis has a socio-economic disparity, as often parents are required to pay for expensive diagnostic testing. Therefore, only those with the means to be tested are able to access the support brought by a diagnosis (Livingston et al., 2018). My own experience echoed this, where those I knew who were diagnosed at a young age were diagnosed privately.

Your background, whether you’re having to care for someone, whether you’re, like, getting extra help or not, whether you’re going to a private school, whether your mum and dad can afford extra tutors… makes such a massive difference. Say you’ve got younger siblings that you have to try and help care for, or you’ve got an elderly… grandparent that you’ve got to look after on your weekends, or… if you’ve got to work at the weekends, those are days that you could be revising... And I think… it’s so unfair that that is not taken into consideration, it’s just did you get your three A’s (grade requirements).

These factors may all contribute to whether or not an individual achieves the required grades. Therefore, candidates’ backgrounds could potentially stop them from considering medicine as a career. It also reiterates that the achievement of these grades does not necessarily reflect a candidate’s true abilities, suggesting that their backgrounds should be considered when evaluating their grades. My experiences in both a comprehensive and grammar school also demonstrated how individuals who perform well academically are more likely to be diagnosed as dyslexic at higher-achieving schools.

Where dyslexia had previously been something that was only considered in those in the lower sets, everyone at this school was very intelligent. As all of the pupils at the school were considered to be of a high intelligence, there was not the same disparity where only those in the lower sets would be considered for dyslexia.

Whilst an individual’s academic struggles are more likely to be noticed in higher-achieving schools through comparison to their peers, the priorities of different schools also likely play a role. My comprehensive school’s goals for exam results were targeted at the maximum number of students passing their basic secondary education. As such, their main focus was on helping those who were likely to fail. However, at my grammar school, their goals were to perform as well as possible in the league tables by helping their students achieve the highest grades possible and get into the best universities. Grammar schools, much like private schools, are therefore more incentivised to test those who may go unnoticed in comprehensive schools to give their students every possible advantage and maintain the school’s reputation. With grammar schools only being available in certain regions of the UK and private schools not being an option for most, this is likely another way in which someone’s socio-economic background may affect the likelihood of entering the medical profession.

### Personal impact of medical school admissions process

I think it definitely added on the pressure, cause it was like, I’ve got to get these three A’s first time

My decision to study medicine impacted my life in several ways during my further education. It created an internal pressure not only to achieve the grades but also to involve myself in extra-curricular activities and seek work experience opportunities. When I performed poorly in an exam, this pressure would only amplify. I would be frustrated with myself that I could not understand, was not working hard enough, or was not smart enough. Especially in my final year, knowing that the final exams were getting ever closer, I felt like I always had too much to learn and not enough time, even when I was months away from the exams.

In maths, I had this mock… I felt like it was just going horrifically wrong… I remember I had my hair down, so that I was covering my face, and I was just crying into this mock, [be]cause… I just couldn’t. I cry when I get frustrated, and the fact that I was just sat there going “I don’t understand it, and there’s nothing I can do here”, I was just… just crying. And I was just writing, some profanities… all over my maths paper because I was just so annoyed.

The pressure I placed on myself, combined with the struggles I was facing academically, impacted me emotionally, making me feel overwhelmed and isolated, even from some of my closest friends.

*I sat in my nice, empty form room trying to do some work. I had gotten the early train in so that I had an hour to myself to revise. But it wasn’t happening. Frustrated, I flipped to another topic in my physics textbook and tried again, trying to focus. I look at my phone and realise my friends will be coming into the station soon. Had they missed me this morning? Feelings of missing my friends and longing for a time when schoolwork didn’t control my life overwhelm me. I can feel the tears coming. I dash for the bathroom and lock myself in a stool as they overwhelm me. I’m not even sure why I’m crying. It just all feels too much. Eventually, I compose myself, splashing my face with cold water to try and make my eyes look less red. Presentable again, I look at the clock. I have a little while left, so I head back off to the books to try desperately to get some work done, because these early mornings, and this loneliness, have to be for something. So, it’s back to the books. (Vignette 4).*

The negative emotions I was feeling made studying harder, creating additional guilt and pressure– a vicious cycle. There is currently no literature documenting the emotional impact of the medical school admissions process on candidates. My experience, however, is undoubtedly one of high pressure and negative emotional impact, worsened by the challenges I experienced due to my undiagnosed, and thus unsupported, dyslexic status.

### What makes a good doctor

*I sat talking to one of my close friends one night. She had always wished to do medicine, but due to her chemistry grades, that was no longer an option to her. As I listened to her discussing how she had no idea what to do now, I felt incredibly sad. She was a kind, empathetic person who would have made a wonderful doctor. But alas, the strict entry requirements and the expense of postgraduate study meant that she would likely never become one. (Vignette 5).*

The topic of selecting the applicants who will become the best doctors has divided opinions for decades (Powis et al., 2019). Is this really with grades and aptitude tests, as the current system suggests (Powis et al., 2019; Hughes, 2002)? A recent study has even identified that characteristics such as gender, ethnicity and confidence level can predict a candidate’s likelihood of having a professionalism issue later in their career (Paton et al., 2018). Based on this, should these characteristics influence whether a candidate is admitted to medical school? Or should the more intangible skills, such as emotional intelligence (Romanelli et al., 2006), communication skills and empathy be considered? After all, these are shown to positively impact patient care (Hughes, 2002).

Some people get like 3A*s and cannot talk to people to save their life. And some people I know who wanted to do medicine, and didn’t get the right grades, and they’re so good at talking to people, they’re such compassionate caring people and I’m like “surely these are the kinds of people that medicine wants to recruit?”. Because, yes, the science is hard, but, I think you can learn it… And, actually, your communication will make… more of a difference to your patients than how well you did in your A-levels.

Whilst a certain level of academic ability is required to study medicine, the current grade requirements may be stopping a multitude of candidates from entering the medical profession, including those undiagnosed as dyslexic and those from disadvantaged backgrounds (Hughes, 2002). Attributes such as being a good communicator and being empathic have been identified as some of the self-reported strengths of dyslexic doctors (Shaw et al., 2016; Shaw & Anderson, 2018). As these are desirable qualities in medical professionals and have been shown to positively impact patient care, barring these candidates based upon grades is potentially barring a cohort that, with the correct level of academic support, would make excellent doctors (Vogel et al., 2018).

Medical schools… need to think about why they’re using the entrance requirements that they’re using and think about who they could be blocking out. It could be that, if they lowered it (the grade requirements), they would get thousands more applications…But… (they) could be getting thousands more applications and actually be choosing better people because of it.

### Undiagnosed dyslexia influencing life course

This autoethnographic process has highlighted how different my life may have been had I received an earlier diagnosis.

When I saw my results, I suddenly knew… I was straight to Google going ‘which med schools will allow you to retake?’ and I found a list of all the entry requirements and who would allow you to retake, and it was UEA, Plymouth and Exeter, and Brighton and Sussex. So, three medical school that would allow you, if you missed it by one grade… But anywhere else you had to have mitigating circumstances. So, yeah, if I’d gone and got a diagnosis then, I probably could have gone ‘on reflection, mitigating circumstances’… I really want to go study in Scotland for five years, and none of them would accept resits, and I was… so sad about that.

My failure to achieve the required grades on the first attempt restricted the number of medical schools I could apply to. Not only did this reduce my chances of being accepted by one of these schools (based upon the idea that the more places you apply, the greater your chance of being accepted), but it also dictated where I could be geographically. All of these restrictions occurred because I failed to achieve the grades by one mark. Does that one mark change what kind of doctor I shall become? If I had received an earlier diagnosis, would I have been able to achieve the grades the first-time round? Would I be studying in Scotland right now? These questions are highly speculative and hypothetical. However, they raise the valid question of whether other undiagnosed dyslexics are having their life courses altered, either geographically or by having their entire career options changed, based upon their failure to achieve the necessary grades.

The UKCAT I hated!... BMAT I absolutely loved… It was just basically… an easy version of GCSE English… science and maths. And that level, like having been at A-level level suddenly felt so easy... And then the UKCAT was the complete opposite…It’s so time pressured… you have to make this decision as to whether you’re going to go at the speed that you can go at, and get the marks, or you’re just going to try and power through as quickly as possible, getting maybe 50% of the marks.

However, even if I had achieved the grades, I likely would not have been admitted to any medical schools using the United Kingdom Clinical Aptitude Test (UKCAT). I did poorly in this exam, achieving a low score which subsequently narrowed my options to a single medical school. This exam format was new and appeared to target some challenges that dyslexic people report. For example, beyond simply being heavily time-pressured, it was conducted on a computer, and a proportion of dyslexic doctors have reported increased difficulty reading from screens, myself included (Anderson & Shaw, 2020). I performed comparatively well in the British Medical Aptitude Test (BMAT), as this was a written exam and, therefore, an exam format I was used to and could use my existing compensatory techniques in. Strong compensatory mechanisms, especially those implemented from childhood, are associated with success in a range of life outcomes in dyslexic adults (Livingston et al., 2018). Undiagnosed dyslexic people applying for medical school likely have academic compensatory techniques in place; therefore, using exam styles like the BMAT may exclude fewer undiagnosed dyslexics from entering the medical profession due to poor aptitude exam scores.

## Meg’s autoethnographic journey

Reflecting upon my experiences has provided new clarity on my emotional response to the admissions process and my further education. By forcing me to acknowledge and process this time in my life, this autoethnography has acted akin to a form of therapy. As I processed my experiences, the emotions I associated with them became less vivid compared to my first recounting, which aided my ability to analyse the results through a more analytic lens. The exploration of the impact of my dyslexia, combined with the immersion in the disability and dyslexia literature, has facilitated a greater understanding of my dyslexia and helped me see the strengths it brings alongside the weaknesses. Overall, this process has been of great personal benefit.

## Strengths and limitations

One of the key strengths of autoethnography, when compared with other ethnographic studies, is that of the researcher’s voice (Lapadat, 2017). Where in ethnography the voices of participants are interpreted and presented by the researcher, in autoethnography, the participant and researcher are one and the same, eliminating the risk of misrepresentation and giving the participant complete control over the narrative being told (Lapadat, 2017). As such, the researcher’s voice is synonymous with the participant’s. However, by the nature of autoethnography, the participant cannot be anonymous, and therefore others who help shape the researcher’s experience are difficult to anonymise, creating a potential dilemma (Lapadat, 2017). Our study abided by relational ethical principles to protect these implicated others.

Due to the focus of autoethnography being on one individuals’ experiences, it is impossible to conclude generalisable results, especially as the relativist ontology states that every individual’s perception of reality is unique (Anderson, 2006). However, Ellis et al. (2011) have argued that, in a sense, generalizability comes from the interpretation and emotional resonance of the readers (Ellis et al., 2011). Furthermore, as an analytic autoethnography, wider theories are incorporated to advance broader sociological knowledge (Anderson, 2006). To achieve this, the researcher must avoid self-absorption, which hinders the production of generalisable theory by failing to understand how their perspective differs from others and the impact this has on the results (Anderson, 2006).

Whilst writing the initial reflective piece, Meg engaged in discussions with others, both from and after this period, using the additional perspectives to enhance the reflective process (Anderson, 2006). The inclusion of an interview held a similar purpose, using an outside perspective to provide a deeper exploration of experiences. However, this could have been further enhanced through discussion with others whose experiences were similar yet unique (Anderson, 2006).

## Conclusions

This autoethnographic study explored the complex impact of Meg’s undiagnosed dyslexic status on her journey to medical school. Her reflections have highlighted the emotional toll that unrecognised dyslexia can have upon an individual when not receiving appropriate support and how this can lead to a sense of isolation. Through this, we have identified how socio-cultural factors might combine with an individual’s unique situation to impact whether undiagnosed dyslexic candidates may successfully gain entry to medical school. Appropriate and early support were identified as critical factors in whether an undiagnosed dyslexic candidate was likely to be successful in their application to medical school, both at an individual and institutional level. Masking, a term most commonly associated with autistic people, was also identified as a potential barrier to diagnosis. The ability to mask was also felt to be dependent on the type of school, with higher-achieving schools potentially introducing more challenges, thus making masking more difficult, alongside a host of wider issues. Finally, we discussed how medical schools need to consider how their current admissions process may be inadvertently disadvantaging these candidates, who have the potential to bring a spectrum of strengths to the profession. There is now a need for further research in this area – perhaps, for example, a more comprehensive phenomenological study of this topic.
